# Community participation and sustainability – evidence over 25 years in the Västerbotten Intervention Programme

**DOI:** 10.3402/gha.v5i0.19166

**Published:** 2012-12-17

**Authors:** Margareta Norberg, Yulia Blomstedt, Göran Lönnberg, Lennarth Nyström, Hans Stenlund, Stig Wall, Lars Weinehall

**Affiliations:** 1 Ageing and Living Conditions Programme, Centre for Population Studies, Umeå University, Umeå, Sweden; 2 Umeå Centre for Global Health Research, Umeå University, Umeå, Sweden; 3 Department of Public Health and Clinical Medicine, Epidemiology and Global Health, Umeå University, Umeå, Sweden

**Keywords:** health surveys, intervention, community participation, primary health care, selection bias

## Abstract

**Background:**

Selection bias and declining participation rates are of concern in many long-term epidemiological studies. The Västerbotten Intervention Programme (VIP) was launched in 1985 as a response to alarming reports on elevated cardiovascular disease (CVD) mortality in Västerbotten County in Northern Sweden. The VIP invites women and men to a health examination and health counselling during the year of their 40th, 50th, and 60th birthdays.

**Objective:**

To evaluate trends in participation rates and determinants of participation in the VIP from 1990 to 2006.

**Design:**

Registry data on socio-economic status from Statistics Sweden, and mortality and hospitalisation data from the National Board of Health and Welfare, both covering the whole Swedish population, were linked to the VIP and analysed for participants and non-participants.

**Results:**

During 1990–2006, 117,710 individuals were eligible to participate in the VIP, and 40,472 of them were eligible to participate twice. There were 96,560 observations for participants and 61,622 for non-participants. The overall participation rate increased from 56 to 65%. Participants and non-participants had minimal differences in education and age. Initial small differences by sex and degree of urban residence decreased over time. Despite an increasing participation rate in all groups, those with low income or who were single had an approximately 10% lower participation rate than those with high or medium-income or who were married or cohabitating.

**Conclusion:**

Sustainability of the VIP is based on organisational integration into primary health care services and targeting of the entire middle-aged population. This enables the programme to meet population expectations of health promotion and to identify high-risk individuals who are then entered into routine preventive health care services. This has the potential to increase participation rates, to minimise social selection bias, and to reinforce other community-based interventions.

Cardiovascular disease (CVD) remains the number one cause of death globally, and the majority of CVD deaths occur in low- and middle-income countries (1, 2). In Scandinavian countries, differences between socio-economic groups are relatively small, yet differences in health are substantial (3). In Sweden, discrepancies in CVD mortality were observed in the early 1980s and remain apparent (4) both between and within regions (5, 6). By the early 1980s, several CVD prevention trials were underway. Examples of such programmes include the North Karelia Project in Finland and the Stanford Five City Multifactor Risk Intervention in the USA. These large-scale, centrally administered programmes combined high-risk and population strategies and were performed mainly outside the health care system.

Responding to alarming reports of elevated CVD mortality, the Västerbotten County Council in northern Sweden decided to launch the Västerbotten Intervention Programme (VIP) in 1985, which included health examinations aimed at reducing CVD (7). This decentralised programme was developed on a small scale in collaboration with researchers, local policy makers, and health care providers, and then was disseminated to the whole County.

Differences between participants and non-participants are of concern in many epidemiological studies and intervention programmes. Such differences may bias population estimates derived from health examinations (8). Generally, non-participants have lower education and lower income, tend to be younger, male, and single, and have poorer health and less healthy life-styles compared to participants (8–11). Moreover, long-term epidemiological studies often experience a decline in participation over time (8, 10, 12, 13).

This paper is part of an on-going evaluation of the VIP. The aim is to analyse trends in participation rates and identify determinants of VIP participation from 1990 to 2006.

## Methods

### The setting and the Västerbotten Intervention Programme

Västerbotten County is located in northern Sweden and has an area of 55,000 km^2^. The population of 260,000 is concentrated on the coast; 45% of the population lives in the city of Umeå, and 28% lives in the town of Skellefteå. The rest are spread across small towns, municipalities, and rural areas. Health care in Sweden is mainly financed by taxes. Each year, around half of the population visit their primary health care (PHC) centre, where 2–10 general practitioners commonly work together and collaborate with district nurses, midwives, physiotherapists, occupational therapists, and social workers. In general, primary prevention is organised at antenatal and child welfare clinics, while systematic preventive programmes that target chronic diseases among adults are lacking.

The VIP is a community intervention that was first developed as a pilot project in the small municipality of Norsjö in 1985. Norsjö was chosen because CVD mortality was highest there. A population strategy with many local community activities and an individual approach were combined. This strategy and the programme were developed in collaboration with the general population and with the involvement of a wide range of sectors in the municipality. All inhabitants were invited to their local PHC centre for a health examination and individual counselling during the year of their 40th, 50th, and 60th birthdays. Thus, as everyone in the population at a certain age point was involved, this was considered to be a community participation approach. Thirty-year olds were also included until 1995. The idea was both to identify the individuals at high risk of CVD and enter them into follow-up and routine preventive health care services, and health promotion that target the entire population within the community. Half of the County's PHC centres joined the programme in 1990 and by 1992 the VIP was working in all the County's municipalities, with health care centres taking responsibility for delivering the programme to their own catchment populations and adapting the activities to fit in well with the services offered at specific health care centres. During the scale-up phase, the programme was discontinued for periods at some PHC centres, for example, if the VIP-nurse was on sick leave, or if they preferred to allocate more resources to other medical issues. Therefore in 1995, the County Council decided to secure access to the VIP for all the middle-aged population by integrating VIP into the basic PHC routines, with PHC being responsible for inviting all eligible subjects to the VIP. The background and design of the VIP is described in detail elsewhere (7).

### The Linnaeus database

This study utilises data from the Linnaeus database (14, 15), that links nationwide registries on socio-economic status (SES) from Statistics Sweden, and mortality and hospitalisation data from the National Board of Health and Welfare to the regional VIP database of 1990–2006.

### Study design and sample

Repeated cross-sectional VIP health surveys were used to estimate participation rates and characteristics of participants versus non-participants during 1990–2006. Individuals registered as living in Västerbotten during the year of their 40th, 50th, and 60th birthdays, totalled 117,710 during this time. Of these, 40,472 were eligible for two VIP surveys and, thus, contributed two observations. The entire 158,182 participant and non-participant observations are included. The population of each municipality was included, starting from the year VIP was introduced into the respective municipality.

### Variables

Data on SES were obtained for the specific year an individual turned 40, 50, or 60 years of age. Medical history data were obtained for the 5 years prior to those same years. The following categories were applied:

*VIP participation*: Participant or non-participant. Participants were individuals who were registered as living in Västerbotten during the year of their 40th, 50th, and 60th birthdays and who participated in the VIP during that or the following year.^1^ Non-participants were those registered at those ages as living in Västerbotten, but who did not participate.

*Country of birth*: Sweden, European Union, or non-European Union.

*Marital status*: Married/cohabiting or single. The category ‘single’ included those who were widowed, divorced or never married. In the national registries, cohabiting couples are registered as cohabiting only if they have children together; otherwise they are listed as single.

*Education*: High (college or higher, ≥13 years of schooling), middle (residential college for adult education or high school, 10–12 years), or low (elementary and comprehensive compulsory school, ≤9 years).

*Individual income*: High (highest quartile of all incomes), middle (second and third quartiles) or low (lowest quartile).

*Residence*: Urban (Umeå, a city with a university and regional administration), semi-urban (Skellefteå, an industrial town; and Lycksele, a small inland town and commercial centre), or rural (smaller towns and villages).

*Number of prior hospitalisations*: 0, 1 or ≥2.

*Prior CVD hospitalisations*: Yes or no. Hospitalisations due to any type of disease of the circulatory system (ICD 10: I10–99 or corresponding ICD 9 diagnoses).

### Statistical methods

STATA software was used for the statistical analyses. The significance level was set at *p*<0.05. Due to the large sample size, all results were statistically significant. SES characteristics of participants and non-participants are provided for the entire follow-up period. For evaluation of trends, the study period was divided into four periods (1990–1994, 1995–1998, 1999–2002, and 2003–2006), and participation rates by background characteristics were assessed for each of the four periods as well as the whole study period. Differences in distributions of characteristics and participation rates are given as absolute percentages. Analyses were carried out for all age groups stratified by sex. To control for age effects, a separate analysis was done for men and women aged 40, 50, and 60 years. Since no differences were found, the results are not shown. Univariable and multivariable logistic regressions were used to estimate odds ratios (ORs) with 95% confidence intervals (CIs) for non-participation versus participation.

## Results

### Overall participation rates in the VIP

**Fig. 1:**
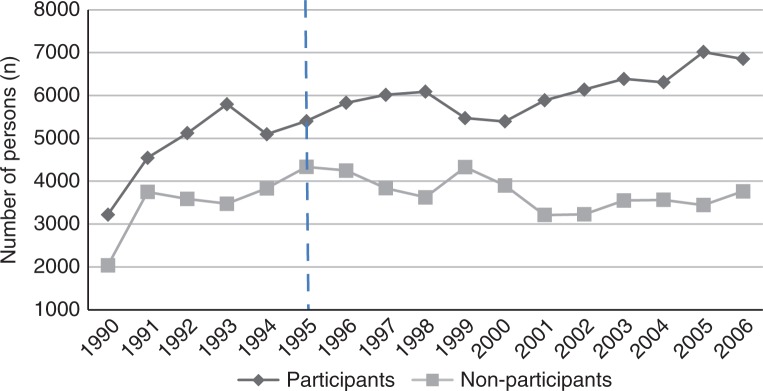
Annual number of participants and non-participants in the Västerbotten Intervention Programme (VIP), 1990–2006. The dashed vertical line indicates 1995, the year the VIP was integrated into basic primary health care services.

The eligible study population increased rapidly until the whole County had joined the VIP by 1992. After that, the increase proceeded at a slower pace and reflected the overall increase of the County's population. Since 1995, about 10,000 individuals annually (range 9,099–10,616) were eligible to be invited to the VIP. During 1990–2006, there were a total of 96,560 participant observations and 61,622 non-participant observations (Fig. 1). The overall participation rate increased from 56% in 1995 to 65% in 2006. Individuals who had participated at least once numbered 78,085 (66%), and 39,625 individuals never participated. Among the 40,472 individuals who were eligible twice, 46% participated twice, 14% only at the initial opportunity, 21% only at the second opportunity, and 19% never participated.

### Characteristics of participants and non-participants

For both men and women, the most pronounced differences between participants and non-participants were that non-participants had an approximately 10% higher proportion of individuals with low income, and an approximately 10% higher proportion with single marital status. Differences in education, age, urbanisation, or country of birth were <5%. During the study period, the proportion of individuals with low education decreased and the number of individuals aged 60 years increased (data not shown).

During the 5-year period preceding the opportunity to be eligible for VIP, hospitalisation for CVD was registered for 5.1% of male and 3.3% of female participants, and 7.5% of male and 5.2% of female non-participants. Nearly 16% of male participants and non-participants had been hospitalised once (all causes). At least two hospitalisations had occurred for 9.3% of male participants and 13.1% of male non-participants. Corresponding numbers among women were single hospitalisations for 20.2% of participants and 20.0% of non-participants. At least two hospitalisations had occurred among 12.9% of female participants and 17.6% of female non-participants.

### Participation rates by background characteristics

The participation rates by background characteristics during the four periods and the overall study period are shown for men and women in Tables 1 and 2. Overall participation rates increased in all groups. The difference between men and women decreased over time, and participation reached 63% among men and 68% among women during 2003–2006. Participation rates increased in the urban and semi-urban areas and was stable in rural areas. This resulted in 2–3% differences in 2003–2006.

**Table 1:** Number of participants and participation rates (%) in the Västerbotten Intervention Programme (VIP), 1990–2006, among men, by background characteristics

		Participation rate				
		
Characteristics	Number	1990–1994	1995–1998	1999–2002	2003–2006	Overall (1990–2006)
Age (yr)						
40	15,674	54.0	51.7	53.9	59.5	54.8
50	16,799	55.2	54.5	59.7	63.4	58.1
60	13,974	54.9	60.8	61.7	64.8	60.9
Country of birth^a^						
Sweden	44,679	55.5	56.1	59.4	63.3	58.6
EU	1,150	40.2	41.8	43.9	55.4	45.2
Non-EU	617	33.2	29.4	35.4	42.5	35.9
Marital status						
Married	34,980	58.3	59.5	62.6	67.3	61.8
Single	11,466	44.2	44.0	48.9	53.1	47.9
Education						
High	9,294	55.6	51.6	57.0	61.6	56.6
Middle	25,724	55.8	56.7	60.0	63.9	59.4
Low	11,428	52.7	54.8	55.1	59.1	54.9
Individual income						
High	18,709	58.6	57.0	61.7	66.5	60.9
Middle	19,159	56.2	57.3	60.8	65.3	60.1
Low	8,578	44.5	48.5	48.2	50.5	48.0
Residence						
Urban	17,106	54.0	46.2	55.6	62.2	54.7
Semi	15,960	48.9	59.3	60.2	61.8	57.6
Rural	13,380	63.0	61.8	59.6	64.1	62.1
Number of prior hospitalisations						
0	34,752	55.5	56.1	59.9	63.8	59.0
1	7,394	56.8	54.8	57.9	61.8	57.7
2+	4,300	47.5	49.6	47.0	52.4	49.0
Prior CVD hospitalisations						
No	44,063	55.1	55.6	59.0	63.2	58.2
Yes	2,383	47.3	48.4	47.2	52.9	49.1
Total	46,446	54.7	55.1	58.3	62.5	57.7

a EU is European Union. Non-EU is non-European Union.

**Table 2:** Number of participants and participation rates (%) in the Västerbotten Intervention Programme (VIP), 1990–2006, among women, by background characteristics

		Participation rate				
		
Characteristics	Number	1990–1994	1995–1998	1999–2002	2003–2006	Overall (1990–2006)
Age (yr)						
40	16,857	62.9	60.3	59.8	64.6	61.9
50	17,941	63.8	64.1	65.6	68.3	65.4
60	15,316	61.9	67.4	66.5	69.7	66.5
Country of birth^a^						
Sweden	47,736	63.6	64.6	64.8	68.1	65.3
EU	1,822	54.3	52.9	55.1	63.2	56.2
Non-EU	556	36.0	33.1	42.1	52.1	43.1
Marital status						
Married	37,753	65.6	66.5	67.3	70.9	67.5
Single	12,361	55.2	55.7	55.6	60.0	56.8
Education						
High	14,356	62.3	59.6	63.6	68.1	63.7
Middle	26,394	65.4	66.1	65.1	68.3	66.3
Low	9,364	59.5	62.9	61.0	62.6	61.2
Individual income						
High	6,675	65.2	62.0	65.7	68.8	65.5
Middle	31,894	66.6	67.7	68.1	71.8	68.5
Low	11,545	52.8	54.6	54.4	58.3	55.1
Residence						
Urban	19,230	62.5	54.7	60.9	67.0	61.4
Semi	17,422	58.1	68.3	67.2	66.6	65.0
Rural	13,462	70.0	70.7	64.4	69.8	68.8
Number of prior hospitalisations						
0	33,522	63.1	65.6	65.8	69.2	66.1
1	10,122	66.1	63.3	63.1	66.6	64.8
2+	6,470	58.7	56.3	55.1	58.2	57.2
Prior CVD hospitalisations						
No	48,461	63.2	64.1	64.5	68.0	65.0
Yes	1,653	56.6	51.2	50.8	56.2	53.8
Total	50,114	62.9	63.6	63.9	67.5	64.5

a EU is European Union. Non-EU is non-European Union.

The majority (94%) of the County's citizens were born in Sweden. Participation rates were highest among native Swedes. However, participation rates increased more markedly among those who were not born in Sweden. Men and women born outside the EU increased their participation rates (9 and 16%, respectively), as did those who were born in the EU (15 and 9%, respectively).

Hospitalisations decreased for the whole population. A quarter of men and a third of women had a history of hospitalisation. Most of them were hospitalised once and participated as frequently as those without prior hospitalisation. Those with at least two hospitalisations had an approximately 10% lower participation rate. Five percent of men and three percent of women had been hospitalised due to CVD. Their participation rates increased slightly among men and reached 53% at the end of the study period. Rates were stable at approximaely 54% among women.

In order to utilise the longitudinal relation between SES characteristics and participation in the VIP, SES data from the year prior to the specific year of VIP eligibility were used and did not change the results (data not shown).

### Logistic regression

Univariable and multivariable logistic regressions found associations between each background characteristic and non-participation versus participation in the VIP that were concordant with participation trends (Table 3). The likelihood of non-participation was highest at the beginning and ORs showed a gradient until the end of the study period. Education and number of hospitalisations prior to the VIP observation had minimal impact on the ORs. Those who were single or had low income were 60% more likely to be non-participants. Non-native Swedes were 90% more likely to be non-participants. Those who were men, younger, lived in semi-urban or urban environments, or who had prior CVD hospitalisations were 40% more likely to be non-participants.

**Table 3:** Odds ratios (ORs) with 95% confidence intervals (CIs) for non-participation in the Västerbotten Intervention Programme (VIP), 1990–2006, by socio-economic characteristics and period. Of the 117,710 individuals eligible for participation during 1990–2006, 40,472 were eligible twice. Thus, there were 158,182 total observations

	Participants			
			
Characteristics	Yes (*n*)	No (*n*)	Univariable model OR (95% CI)	Multivariable model OR (95% CI)
Sex				
Women	50,114	27,558	1	1
Men	40,446	34,064	1.33 (1.31–1.36)	1.37 (1.34–1.40)
Age (yr)				
40	29,289	16,692	1	1
50	34,740	21,615	1.09 (1.06–1.12)	1.23 (1.20–1.26)
60	32,531	23,315	1.26 (1.23–1.29)	1.42 (1.38–1.46)
Country of birth^a^				
Sweden	92,415	56,975	1	1
EU	2,972	2,810	1.53 (1.46–1.62)	1.39 (1.31–1.46)
Non-EU	1,173	1,837	2.54 (2.36–2.74)	1.90 (1.76–2.05)
Marital status				
Married	72,733	39,761	1	1
Single	23,827	21,861	1.68 (1.64–1.72)	1.60 (1.57–1.64)
Education				
High	23,650	15,317	1	1
Middle	52,118	30,996	0.92 (0.90–0.94)	0.89 (0.87–0.91)
Low	20,792	15,309	1.13 (1.10–1.17)	1.05 (1.02–1.09)
Individual income				
High	25,384	15,551	1	1
Middle	51,053	27,361	0.87 (0.85–0.90)	0.98 (0.96–1.01)
Low	20,123	18,710	1.52 (1.48–1.56)	1.60 (1.55–1.65)
Residence				
Urban	26,842	14,272	1	1
Semi	33,382	21,104	1.19 (1.16–1.22)	1.25 (1.21–1.28)
Rural	36,336	26,246	1.36 (1.32–1.39)	1.38 (1.34–1.41)
Number of prior hospitalisations				
0	68,274	41,385	1	1
1	17,516	10,925	1.03 (1.00–1.06)	0.98 (0.95–1.01)
2+	10,770	9,312	1.43 (1.38–1.47)	1.18 (1.14–1.22)
Prior CVD hospitalisations				
No	92,524	57,728	1	1
Yes	4,036	3,894	1.55 (1.48–1.62)	1.35 (1.28–1.42)
Time period				
2003–2006	26,559	14,320	1	1
1999–2002	22,889	14,579	1.18 (1.15–1.22)	1.19 (1.16–1.22)
1995–1998	23,336	16,042	1.27 (1.24–1.31)	1.28 (1.25–1.32)
1990–1994	23,776	16.681	1.30 (1.26–1.34)	1.32 (1.28–1.36)

a EU is European Union. Non-EU is non-European Union.

## Discussion

The purpose of the VIP was to conduct a community intervention to reduce CVD that would reach the entire middle-aged population in Västerbotten County. The main finding of this study is that the VIP coverage has continuously increased among all groups, regardless of socio-economic characteristics, age, sex, or medical history. This is contrary to most other epidemiological studies (8, 10, 12, 13, 16, 17). In the Northern Sweden WHO-MONICA project, on-going in the same region as the VIP, there was a decrease in participation from 81.2% in 1986 to 67.4% in 2009 for ages 25–64 years (13). Similar trends are observed in other MONICA sites (8), Denmark (10), and Scotland (17). The North Karelia project in Finland was a model for the VIP; preventive activities, including health examinations, were gradually developed and new survey areas were added. However, participation rates have declined at all Finnish survey sites (12). The continuous increase in the VIP participation rate indicates the potential for a sustainable PHC-based intervention to reach the majority of the population. This is also supported by the fact that out of those who were eligible to participate in the VIP twice, 81% participated at least once. Recent administrative reports from the Västerbotten County Council, which showed a continuing increase in participation rates to 69% in 2010 and 73% in 2011, further emphasise this point. These data were not used in the results section because they were not available in the Linnaeus database, which only links data up until 2006. The recommended level of 70% participation rate for population studies (18, 19) was not reached until recent years; therefore, prevalence estimates should be taken with caution.

Several factors may have contributed to the programme's sustainability and increasing participation trends. Firstly, the VIP is organised and delivered within an established and stable structure, which is the routine PHC. The vast majority of the population is familiar with and has high confidence in PHC in Sweden. In contrast, other surveys often are performed by specific research organisations, although some Finnish surveys were carried out in local health centres by specially trained nurses (12). In the Netherlands, a higher participation rate also was observed when general practitioners had an active role (20). Second, the VIP was planned in collaboration with local stakeholders and the general population, and is a good example of community participation. This was most evident during the early phases. Nevertheless, the programme continuously targets the whole population at the ages of 40, 50, and 60 years, and the results are also fed back to local community leaders to be an integrated part of health promoting community activities. This is in contrast to other surveys that periodically invite random samples and do not have any direct link to the local community. According to local public opinion, the VIP is increasingly perceived as everyone's right. Eligible VIP participants often request a VIP appointment themselves. The parallel with preventive child and maternity health care services, which are organised within PHC, is obvious. Third, while other surveys primarily collect data for epidemiological research, the VIP is designed to meet population expectations on health promotion with a dual strategy: 1) individual health counselling for all participants that complements community interventions; and 2) identification of high-risk individuals who are cared for within the same organisation. Data from the VIP are regularly also fed back to local policy makers to be an integral part and linked into community activities for the population in each municipality. Fourth, determined support from the political and decision-making structures at the Västerbotten County Council level, as well as the VIP organisation with continuous updating of routines, support, education, and feedback to the VIP-nurses, should be acknowledged as a strong basis for stability and sustainability.

This study found increasing similarities in participation rates among men and women and in urban and rural areas. However, similar to other epidemiological studies (8–11, 13, 21), differences were observed between participants and non-participants that suggest social selection bias. Compared with other studies performed in Sweden (13, 22), we conclude that overall disparities in the VIP are smaller with regard to education, but similar for sex, marital status, age, and country of origin. The present results are in accord with a previous evaluation of the 1992–1993 VIP, which concluded that differences were only marginal in age, education, and socio-economic class based on profession (23). Organised screening programmes for breast and cervical cancer have been favoured for opportunistic screening among women with low educational levels and low occupational class (24). Yet, it is plausible that the VIP, nested within the PHC organisation with its systematic approach, not only contributes to higher participation rates but also limits socio-economic differences between participants and non-participants.

Despite increasing participating rates among those with the lowest income or those who live alone, it remains a challenge for the VIP to reach these groups. For example, those with severe mental illness often have low incomes because of lower working capacity. This group is at particularly high risk of CVD (25) and is probably overrepresented among non-participants (26). Health behaviour is strongly related to educational level (22, 27–29); therefore, similar participation rates, regardless of education, are a prerequisite if the VIP is to contribute to improved public health.

Other studies show that participants may have worse health (20, 30), similar health (21, 31) or, most frequently, better health (11, 32, 33) or utilise more PHC but less hospital care (34) compared to non-participants. In the VIP, history of CVD hospitalisations increased the likelihood of non-participation. This might partly be due to low motivation for a health examination among individuals who already have their risk factors or disease monitored and controlled. Concordant with our study, a Danish-registry-based study (10) showed higher hospital admissions rates among non-participants, but only during a limited period before and during the survey. We did not investigate the time point at which the hospitalisations ocurred during the 5 years before the VIP.

### Strengths and limitations

The Linnaeus database allows us to use reliable registry data instead of self-reported data or a selected sample of the resource population when comparing participants and non-participants. Although participants and non-participants were similar in socio-economic characteristics and number of previous CVD hospitalisations, we could not control for discrepancies in life style. Although the similarity in education indicates that this should not create significant bias, the issue of selection bias remains difficult to explore fully. One consequence of VIP's integration into primary care is that other, more urgent medical needs might be periodically prioritised; therefore, VIP invitation rates are not always 100%. In 2002, the County Council sent a postal questionnaire to those who were eligible for VIP participation during 2001 (*n*=9,159). The response rate was 89%; 65% had participated, 5% wished for more information, 5% were already under medical care, 1% did not want to participate, 5% had forgotten the appointment, and 8% responded that they had not been invited to the VIP (not published). This means that non-participation rates include both refusals and not being invited. Not being invited does not confer selection bias; therefore, this might contribute to the socio-economic differences between participants and non-participants being small.

## Conclusions

Participation rates in the VIP increased from 56% in 1995 to 65% in 2006, and 81% of those who were eligible twice at 10-year intervals participated at least once. Increasing participation rates occurred regardless of socio-economic characteristics, age, sex or medical history. This is unique. Although a challenge remains to reach those who have low income, live alone or are born outside Sweden, background characteristics were similar and changed similarly in both participants and non-participants during the study period of 1990–2006. This is most likely due to sustainability, which is based on integration of the VIP into the structure and organisation of PHC services, as well as targeting the entire middle-aged population. Because of these characteristics, the VIP is able to meet population expectations on health promotion and has the potential to identify individuals at high risk of CVD and smoothly pass them into ordinary preventive measures, as well as to reinforce community-based interventions for improved public health.

## Footnotes

1 Individuals who could not participate in the VIP during the year of their 40th, 50th, and 60th birthdays but were able to participate at the beginning of the next year, were categorised as VIP participants in the year of their birthday.
